# Mapping the applications of stable isotope analysis in rodent ecology: Protocol for a scoping review

**DOI:** 10.1371/journal.pone.0361880

**Published:** 2026-11-13

**Authors:** Barbara Protocevich, Jackson Epaminondas de Sousa, Rafael Duarte Paes, Ana Clara Gomes Silva, David Miguel Vieira Filipe, Geovanne Augusto Cantuária, Maria Eduarda Pereira de Souza, Maria Eduarda Bassan Biscaro, Samanta Helena Martins de Oliveira, Mabel Fernandes Figueiró, Luciane Cruz Lopes, Thiago Simon Marques

**Affiliations:** 1 Universidade de Sorocaba – UNISO, Sorocaba, Brazil; 2 Universidade Federal de São Carlos – USFCAR, Sorocaba, Brazil; 3 HCor – Hospital do Coração, São Paulo, Brazil; National Museums of Kenya, KENYA

## Abstract

**Background:**

Rodents (order Rodentia) are the most diverse and geographically widespread order of mammals, occupying virtually all terrestrial ecosystems and playing key ecological roles in food webs and ecosystem functioning. Stable isotope analysis has become a tool for investigating trophic ecology, resource use, habitat use, and movement across spatial and temporal scales. Although isotope applications in rodent ecology have expanded in recent decades, the literature remains diverse, and no comprehensive synthesis currently maps this evidence. This scoping review aims to systematically map and synthesize how stable isotope analysis has been applied in ecological studies of the order Rodentia across diverse ecosystems.

**Methods:**

This protocol follows the methodology of the Joanna Briggs Institute (JBI), Adelaide, South Australia for scoping reviews and will be reported in accordance with PRISMA-ScR guidelines. Searches will be conducted in Scopus, Web of Science, PubMed, SciELO, and Google Scholar (first 200 results sorted by relevance), covering studies from database inception to January 2026. Eligible sources will include peer-reviewed articles, master’s theses, and doctoral dissertations published in any language, searches will be conducted in ProQuest, BTDT (Biblioteca digital brasileira de teses e dissertações) and CAPES Theses and Dissertations. Nine reviewers will screen randomized titles and abstracts in pairs, with a pilot-test of 100 random articles to ensure reviewers consistency. Full-text assessment and data charting will be conducted by the reviewer pairs using a standardized form, with a pilot-test verification of a subset of included studies to ensure consistency. No critical appraisal will be performed.

**Discussion:**

This review will provide a structured overview of stable isotope applications in rodent ecology, identifying methodological patterns, ecosystem and geographic coverage, and knowledge gaps to inform future research and evidence syntheses.

**Registration:**

This protocol has been registered in the Open Science Framework (OSF) 10.17605/OSF.IO/GQW3B.

## Introduction

Rodents (order Rodentia) constitute the most diverse and geographically widespread order of mammals, occupying virtually all terrestrial ecosystems [1]. Their global distribution, high abundance, ecological plasticity, and broad range of trophic strategies position rodents as key components of food webs and ecosystem functioning across environmental gradients [2,3]. As primary consumers, omnivores, and prey, rodents play a central role in energy transfer, nutrient cycling, and the structuring of ecological communities [1].

Conventional approaches to assessing rodent diets, such as stomach content and faecal analyses, capture only short-term dietary information and are difficult to standardize across studies. Stable isotope analysis offers an integrative alternative by reflecting assimilated resources over extended temporal scales [4,5]. The fundamental principle underlying this approach is that the isotopic composition of consumer tissues reflects that of assimilated resources in predictable ways, allowing dietary and ecological inferences over extended temporal scales [6–9]. Although stable carbon (δ^13^C) and nitrogen (δ^15^N) isotopes are the most commonly applied tracers in rodent studies—providing insights into basal resource use, primary production pathways, and trophic position [10,11]—other isotope systems have also been employed. Stable hydrogen (δ^2^H) and oxygen (δ^18^O) isotopes have been used to investigate spatial origin, water sources, movement, and climatic influences on mammalian tissues, including rodents [12–14]. Additionally, stable sulfur (δ^34^S) isotopes have contributed to distinguishing among food webs influenced by marine, freshwater, terrestrial, or anthropogenic sulfur sources, particularly in systems where carbon and nitrogen alone provide limited resolution [15,16].

Within the order Rodentia, these multiple stable isotopic systems have been applied across diverse ecological contexts, including natural ecosystems, agroecosystems, and urban environments, enabling assessments of resource partitioning, niche breadth, habitat connectivity, and responses to environmental gradients [17–19]. However, the application of hydrogen, oxygen, and sulfur isotopes to rodent studies remains less standardized and less frequently synthesized compared to carbon and nitrogen.

A scoping review is particularly appropriate for mapping heterogeneous evidence, clarifying the extent and characteristics of available literature, and identifying knowledge gaps. In brief, a systematic review addresses a narrowly defined question by exhaustively and critically appraising a delimited body of evidence, typically to inform a specific practice or policy decision, whereas a scoping review maps the breadth, nature, and extent of a more heterogeneous literature, clarifies key concepts, and identifies knowledge gaps, without formally appraising study quality [19,20]. Although individual studies and narrative discussions exist, a structured synthesis of methodological approaches, isotope systems, and ecological contexts remains lacking. To our knowledge, no previous scoping review or systematic review has comprehensively mapped the application of stable isotope analysis in the ecology of the order Rodentia across ecosystems. This gap persists despite broader, more recent syntheses of stable isotope methodology and isotopic-niche concepts across taxa, such as the updated review by Shipley and Matich [21], none of which has focused specifically on Rodentia.

Given this diversity and dispersion of evidence, a scoping review represents the most appropriate approach to systematically map and organize the available literature. By compiling studies that apply stable isotope analysis to the order Rodentia across different ecosystems and methodological designs, this scoping review aims to identify major research trends and analytical approaches, reveal underexplored geographic regions, ecological contexts, and research questions, and highlight knowledge gaps that may guide future systematic reviews and targeted experimental studies.

Importantly, this synthesis will integrate studies employing multiple stable isotope systems that include carbon, nitrogen, hydrogen, oxygen, and sulfur, thus allowing a broader evaluation of how stable isotopic tools have been used to investigate trophic dynamics, resource use, movement, and ecosystem connectivity in rodents. By providing a structured overview of isotope applications within the order Rodentia, this scoping review will contribute to advancing the understanding of rodent ecological roles across diverse landscapes and will offer a conceptual and methodological framework to support future research, conservation planning, and the application of stable isotopes in ecological monitoring at regional and global scales.

## Review question

In order to map and synthesize the applications of stable isotope analysis in the ecology of the order Rodentia across diverse ecosystems, identifying the stable isotope systems employed, methodological approaches, ecological contexts, and research objectives, as well as highlighting methodological trends, knowledge gaps, and priorities for future research, the following question was posed:

How has stable isotope analysis been applied in ecological studies of the order Rodentia across different ecosystems?

## Eligibility criteria (Population, Concept, and Context [PCC] Framework)

Eligibility criteria for the scoping review was based on the Population (P), Concept (C), and Context (C) (i.e., PCC) Framework [18] as follows:

**Population (P):** Studies involving species belonging to the order Rodentia.

**Concept (C):** Application of stable isotope analysis to investigate ecological processes, including trophic position, diet composition, resource use, niche breadth, habitat use, movement, and environmental integration; and

**Context (C):** All terrestrial ecosystems worldwide, including natural, semi-natural, agricultural, and urban environments.

**Inclusion criteria:**

Inclusion criteria for scoping review included the following:

- Empirical studies (field-based, observational, or conducted under semi-natural conditions) that apply stable isotope analysis to investigate ecological aspects of the order Rodentia;
- Studies reporting quantitative stable isotope data, such as isotopic ratios, means, ranges, variance, niche metrics, or outputs from isotope-based models;
- Studies employing any stable isotope system, including but not limited to carbon, nitrogen, hydrogen, oxygen, sulfur, or other isotopes; and
- Eligible sources include peer-reviewed journal articles, theses, and dissertations.
- Systematic reviews and meta-analyses will be screened only to identify additional relevant primary studies, but will not be included in the synthesis.

**Exclusion criteria:**

Exclusion criteria for scoping review included the following:

- Studies available only as abstracts, conference proceedings, summaries, or extended abstracts that do not provide sufficient data;
- Studies focusing exclusively on health-related or physiological inferences (e.g., microbiota, cellular, enzymatic, or molecular studies);
- Archaeological or palaeoecological studies;
- Studies conducted exclusively under captive or laboratory conditions;
- Narrative reviews, opinion papers, editorials, and methodological papers lacking empirical stable isotope data on the order Rodentia; and
- Studies focusing on mammalian orders other than the order Rodentia or on non-mammalian taxa.

## Methods

### Protocol design

This scoping review follows the Joanna Briggs Institute (JBI) methodology [18] and will be reported in accordance with PRISMA-ScR guidelines [20]. Registered in Platform OSF [19].

As this review uses published and publicly available data, ethical approval was not required. Findings will be disseminated through peer-reviewed publication and scientific presentations.

### Information sources

Searches will be conducted in Scopus (https://www.scopus.com/), Web of Science (https://www.webofscience.com/), PubMed (https://pubmed.ncbi.nlm.nih.gov/), Google Scholar (https://scholar.google.com/) and SciELO (https://www.scielo.org/), covering studies published from database inception to January 2026. Search strategies will use Boolean combinations of terms related to stable isotope analysis, the order Rodentia, and ecological contexts. The primary search strategy will be conducted in English. Studies retrieved in other languages will also be considered during the screening process when identified through the database searches. The complete search strategies and database-specific adaptations are presented in Appendix I.

For Google Scholar, only the first 200 results sorted by relevance will be screened. This approach was adopted to ensure feasibility while maintaining transparency and reproducibility. Gray literature, including theses and dissertations, will also be identified through Google Scholar searches, ProQuest (https://about.proquest.com/pt-br/products-services/pqdtglobal/), BTDT (Biblioteca digital brasileira de teses e dissertações) (https://bdtd.ibict.br/), and CAPES Theses and Dissertations Catalog (https://catalogodeteses.capes.gov.br/catalogo-teses/), and will be recorded as such during the data extraction process.

### Selection process

All records identified through the database searches will be imported into Zotero version 9.0 (https://www.zotero.org/) and the web-based software Rayyan [22,23], where duplicate records will be removed. The selection process will follow the methodological guidance for scoping reviews developed by the Joanna Briggs Institute (JBI) [18]. Nine reviewers will independently screen randomized titles and abstracts in pairs according to the predefined eligibility criteria. The use of reviewer in pairs is intended to reduce the likelihood of selection errors and ensure consistent application of the eligibility criteria [18]. Disagreements will first be resolved through discussion and, when necessary, by consultation with an additional reviewer [18].

Prior to formal screening, the reviewers will pilot-test the eligibility criteria using a random sample of 100 records. This calibration exercise was designed to ensure that reviewers have a consistent understanding and application of the predefined eligibility criteria before commencing the formal screening process. Inter-reviewer agreement may be assessed using percentage agreement as a descriptive measure of consistency during this pilot phase.

Full-text screening will also be conducted independently by reviewer pairs, following the same predefined eligibility criteria and JBI methodological guidance [18]. Disagreements will be resolved through discussion and, when necessary, consultation with and additional reviewer. Reasons for exclusion at the full-text stage will be documented and reported in accordance with the PRISMA-ScR guidance [20]. The overall study selection process will be presented using the PRISMA-ScR flow diagram [20].

### Data extraction

Data extraction will be conducted using a standardized charting form (provided as Supporting Information, S1 File – Data charting form) developed for this review. The charting form will be pilot-tested on a subset of included studies and refined iteratively prior to full data extraction to ensure consistency.

Extracted variables will include geographic location, ecosystem type, species, sample size, tissue type, isotope systems analyzed, analytical approach, ecological focus, study design and key findings. Additional variables may be incorporated during the charting process if considered relevant to the objectives of the review. If necessary, database searches may be rerun after a predefined period of one year to identify newly indexed studies relevant to the review.

### Data analysis and presentation

Data will be summarized descriptively using tables, figures and narrative synthesis. Included studies will be categorized according to stable isotope system, ecological application, ecosystem type, geographic distribution, tissue type, and methodological approach to identify major research trends and knowledge gaps.

### Critical appraisal of individual sources of evidence

Consistent with scoping review methodology, no formal critical appraisal of methodological quality or risk of bias will be conducted. The aim of this review is to map and characterize the available evidence regarding the application of stable isotope analysis in ecology of the order Rodentia rather than to evaluate the quality or certainty of evidence.

## Amendments

Any amendments to this protocol will be documented in the Open Science Framework (OSF) registration and clearly described in the final published review [24].

## Protocol stage statement

At the time of protocol submission, full-text screening, data extraction, and synthesis had not yet commenced.

## Declaration of AI

During the preparation of this work, the author(s) used ChatGPT 4.0 for proofreading and improving the coherence of the written text. After using this tool, the author(s) reviewed and edited the content in accordance with the scientific method and assume full responsibility for the content of the publication.

### Appendix I – Search strategy*

**Table:** 

Database	Query
PubMed	(((“rodentia”[MeSH Terms]) OR (rodentia[Title/Abstract] OR rodent*[Title/Abstract])) AND (“isotopes”[MeSH Terms] OR “stable isotope*”)) AND (“ecology”[MeSH Terms] OR Ecology[Title/Abstract] OR ecosystem*[Title/Abstract] OR “Trophic Niche”[Title/Abstract] OR “Trophic ecology”[Title/Abstract] OR “diet”[MeSH Terms] OR Diet[Title/Abstract] OR Dietary[Title/Abstract] OR “resource use”[Title/Abstract] OR “resource partitioning”[Title/Abstract])
Scopus	TITLE-ABS-KEY ((Rodentia OR Rodents OR Rodent) AND (“stable isotope*” OR Stable W/3 Isotope* OR isotopes)) (ALL(rodentia) OR ALL(rodent)) AND (ALL(“stable isotope”) OR ALL(“stable W/3 isotopes”) OR ALL(isotopes)) AND ALL(ecology)
Web of Science	(ALL=(Rodentia OR Rodents OR Rodent)) AND ALL=(“stable isotopes” OR “Stable W/3 Isotopes” OR isotopes)
SciELO	((((TS=(Rodentia)) OR TS=(rodents)) OR TS=(rodent)) AND TS=(stable isotopes)) OR TS=(isotopic analysis)
Google Scholar	“stable isotopes” AND Rodentia AND (ecology OR diet OR trophic OR niche)

*Two complementary search strategies were conducted in Scopus to maximize sensitivity and capture variations in indexing fields. The first strategy targeted title, abstract, and keywords (TITLE-ABS-KEY), while the second strategy used the ALL field to broaden retrieval.

**Note:** Search strategies will be tailored to each database syntax.

## Supporting information

S1 File Data charting form. (DOCX)

S2 File PRISMA-P Checklist. (DOCX)
