# Supplementary material for: Mapping the applications of stable isotope analysis in rodent ecology: Protocol for a scoping review
Source: PLoS One. 2026 Nov 13;21(11):e0361880. doi: 10.1371/journal.pone.0361880 (PMC13766249; doi:10.1371/journal.pone.0361880)
Supplement: Supplementary file 1 [file pone.0361880.s001.docx]

**S1 File – Data charting form***

| Field | Description / Guidance for Data Extraction |
| --- | --- |
| 1. Reference ID | Unique identifier for each study (e.g., author_year_journal). |
| 2. Author(s) | Full list of authors. |
| 3. Year of Publication | Year the study was published. |
| 4. Title of Study | Full title as published. |
| 5. Country / Region | Geographic location of the study area. |
| 6. Ecosystem Type | Forest, savanna, agricultural mosaic, plantation, urban area, etc. |
| 7. Objective(s) of the Study | Briefly describe the main research question or purpose. |
| 8. Target Species / Taxonomic Information | Specify Rodentia species (scientific name and common name if available). |
| 9. Sample Size | Number of individuals or samples analyzed. |
| 10. Tissue Type Analyzed | Hair, muscle, liver, whiskers, feces, etc. |
| 11. Stable Isotopes Used | δ¹³C, δ¹⁵N (or others if applicable). |
| 12. Analytical Approach / Methodology | Laboratory methods, mixing models, statistical analyses, or isotopic baselines used. |
| 13. Main Ecological Focus | Diet composition, trophic position, habitat use, isotopic niche width, occupation, or temporal variation. |
| 14. Key Findings | Summary of major ecological or methodological results. |
| 15. Limitations / Gaps Identified | Reported limitations or data gaps noted by the authors. |
| 16. Study Design | Observational, experimental, descriptive, comparative, etc. |
| 17. Type of Environment | Field study or semi-natural. |
| 18. Data Source Type | Journal article, master’s thesis, or doctoral dissertation. |
| 19. Funding Source / Acknowledgments | Mention if the study indicates funding related to ecology or isotopic research. |
| 20. Reviewer’s Notes | Space for observations or comments from the reviewer (e.g., relevance, inclusion decision, additional notes). |
| 21. Keywords | Keywords that define the center of the study. |

*****Data extraction will be guided by this standardized form, which may be refined iteratively during the review process to ensure that all relevant information is captured according to JBI methodology**.

**See: Peters MD, Marnie C, Tricco AC, Pollock D, Munn Z, Alexander L, et al. Updated methodological guidance for the conduct of scoping reviews. *JBI Evidence Synthesis.* 2020;18(10):2119–26. <https://doi.org/10.11124/jbies-20-00167>
