# Supplementary material for: Mapping the applications of stable isotope analysis in rodent ecology: Protocol for a scoping review
Source: PLoS One. 2026 Nov 13;21(11):e0361880. doi: 10.1371/journal.pone.0361880 (PMC13766249; doi:10.1371/journal.pone.0361880)
Supplement: Supplementary file 2 [file pone.0361880.s002.docx]

**Preferred Reporting Items for Systematic review and Meta-Analysis Protocols (PRISMA-P) 2015 checklist*: recommended items to address in a systematic review protocol****

| Section and topic | Item No | Checklist item |
| --- | --- | --- |
| ADMINISTRATIVE INFORMATION | | |
| Title: |  |  |
| Identification | 1a | **Mapping the applications of stable isotope analysis in rodent ecology: protocol for a scoping review** |
| Update | 1b | N/A |
| Registration | 2 | In accordance with the guidelines, our scoping review protocol was registered in the Open Science Framework (OSF) on 10 February 2026 and was last updated on 18 May, 2026 (registration number [10.17605/OSF.IO/GQW3B](https://doi.org/10.17605/OSF.IO/GQW3B)). |
| Authors: |  |  |
| Contact | 3a | Barbara Protocevich^a^ (e-mail: [barbarappina@gmail.com](mailto:barbarappina@gmail.com))  Jackson Epaminondas de Sousa^a^ (e-mail: [jackson.sousa@ueap.edu.br](mailto:jackson.sousa@ueap.edu.br))  Rafael Duarte Paes^a^ (e-mail: [r.dpaes@yahoo.com.br](mailto:r.dpaes@yahoo.com.br))  Ana Clara Gomes Silva^a^ (e-mail: [pcclara86@gmail.com](mailto:pcclara86@gmail.com))  David Miguel Vieira Filipe^a^ (e-mail: [dmvfilipe@gmail.com](mailto:dmvfilipe@gmail.com))  Geovanne Augusto Cantuária^a^ (e-mail: [cantuariageovanne@gmail.com](mailto:cantuariageovanne@gmail.com))  Maria Eduarda Pereira de Souza^a^ (e-mail: [mariaedpereirasouza@gmail.com](mailto:mariaedpereirasouza@gmail.com))    Maria Eduarda Bassan Biscaro^b^ (e-mail: [mariabiscaro@estudante.ufscar.br](mailto:mariabiscaro@estudante.ufscar.br))  Samanta Helena Martins^a^ (e-mail: [saamantahelena2018@gmail.com](mailto:saamantahelena2018@gmail.com))  Mabel Fernandes Figueiró^c^ (e-mail: [mabelfigueiro@gmail.com](mailto:mabelfigueiro@gmail.com))  Luciane Cruz Lopes^a^ (e-mail: [luciane.lopes@prof.uniso.br](mailto:luciane.lopes@prof.uniso.br))  Thiago Simon Marques^a^* (e-mail: thiago.marques@prof.uniso.br)  Affiliations  ^a^Universidade de Sorocaba – UNISO, Sorocaba, Brazil  ^b^Universidade Federal de São Carlos – USFCAR, Campus Sorocaba, Brazil  ^c^HCor – Hospital do Coração, São Paulo, Brazil  Corresponding author’s email address  t[hiago.marques](mailto:Luciane.lopes@uniso.br)@prof.uniso.br (T. S. Marques)  Universidade de Sorocaba – UNISO, Sorocaba, Brazil  Rodovia Raposo Tavares, KM 92,5 – Sorocaba, São Paulo, Brazil  ZIP Code: 18023-000 |
| Contributions | 3b | BP conceived and designed the study. All authors contributed to the development of the selection criteria, the risk of bias assessment strategy and data extraction criteria. LCL, TMS and MF contributed to methodological refinement and supervision of the study. All authors reviewed and approved the final manuscript. |
| Amendments | 4 | Any amendments to this protocol will be documented in the Open Science Framework (OSF) registration and clearly described in the final published review. |
| Support: |  |  |
| Sources | 5a | This study is part of the master’s project of Barbara Protocevich, and is funded by the Fundação de Amparo à Pesquisa do Estado de São Paulo (FAPESP) (Grant no. 2024/11011-8). |
| Sponsor | 5b | Fundação de Amparo à Pesquisa do Estado de São Paulo (FAPESP) |
| Role of sponsor or funder | 5c | Scholarship funding |
| INTRODUCTION | | |
| Rationale | 6 | To our knowledge, no previous scoping review or systematic review has comprehensively mapped the application of stable isotope analysis in the ecology of Rodentia across ecosystems. Although individual studies and narrative discussions exist, a structured synthesis of methodological approaches, isotope systems, and ecological contexts remains lacking.  Given this diversity and dispersion of evidence, a scoping review represents the most appropriate approach to systematically map and organize the available literature. By compiling studies that apply stable isotope analysis to Rodentia across different ecosystems and methodological designs, this review aims to identify major research trends and analytical approaches, reveal underexplored geographic regions, ecological contexts, and research questions, and highlight knowledge gaps that may guide future systematic reviews and targeted experimental studies.  Importantly, this synthesis will integrate studies employing multiple isotope systems, including carbon, nitrogen, hydrogen, oxygen, and sulfur, allowing a broader evaluation of how isotopic tools have been used to investigate trophic dynamics, resource use, movement, and ecosystem connectivity in rodents. By providing a structured overview of isotope applications within the order Rodentia, this scoping review will contribute to advancing the understanding of rodent ecological roles across diverse landscapes and will offer a conceptual and methodological framework to support future research, conservation planning, and the application of stable isotopes in ecological monitoring at regional and global scales. |
| Objectives | 7 | To map and synthesize the applications of stable isotope analysis in the ecology of the order Rodentia across diverse ecosystems, identifying the isotope systems employed, methodological approaches, ecological contexts, and research objectives, as well as highlighting methodological trends, knowledge gaps, and priorities for future research.  How has stable isotope analysis been applied in ecological studies of the order Rodentia across different ecosystems? |
| METHODS | | |
| Eligibility criteria | 8 | **Population (P):** Studies involving species belonging to the order Rodentia.  **Concept (C):** Application of stable isotope analysis to investigate ecological processes, including trophic position, diet composition, resource use, niche breadth, habitat use, movement, and environmental integration.  **Context (C):** All terrestrial ecosystems worldwide, including natural, semi-natural, agricultural, and urban environments.  **Inclusion Criteria:**   - Empirical studies (field-based, observational, or conducted under semi-natural conditions) that apply stable isotope analysis to investigate ecological aspects of the order Rodentia. - Studies reporting quantitative stable isotope data, such as isotopic ratios, means, ranges, variance, niche metrics, or outputs from isotope-based models. - Studies employing any stable isotope system, including but not limited to carbon, nitrogen, hydrogen, oxygen, sulfur, or other isotopes. - Eligible sources include peer-reviewed journal articles, theses, and dissertations. - Systematic reviews and meta-analyses will be screened only to identify additional relevant primary studies, but will not be included in the synthesis.   **Exclusion Criteria:**   - Studies available only as abstracts, conference proceedings, summaries, or extended abstracts that do not provide sufficient data. - Studies focusing exclusively on health-related or physiological inferences (e.g., microbiota, cellular, enzymatic, or molecular studies). - Archaeological or palaeoecological studies. - Studies conducted exclusively under captive or laboratory conditions. - Narrative reviews, opinion papers, editorials, and methodological papers lacking empirical stable isotope data on Rodentia. - Studies focusing on mammalian orders other than Rodentia or on non-mammalian taxa. |
| Information sources | 9 | Searches will be conducted in Scopus, Web of Science, PubMed, Google Scholar and SciELO, covering studies published from database inception to January 2026. Search strategies will use predefined Boolean combinations of terms related to stable isotope analysis, the order Rodentia, and ecological contexts. The primary search strategy will be conducted in English. Studies retrieved in other languages will also be considered during the screening process when identified through the database searches. The complete search strategies and database-specific adaptations are presented in Appendix I.  For Google Scholar, only the first 200 results sorted by relevance will be screened. This approach was adopted to ensure feasibility while maintaining transparency and reproducibility, as recommended in scoping review methodology. Gray literature, including theses and dissertations, will also be identified through Google Scholar searches, Proquest and CAPES dissertation and thesis portal and will be recorded as such during the data extraction process. |
| Search strategy | 10 | Database \| Query; PubMed \| ((("rodentia"[MeSH Terms]) OR (rodentia[Title/Abstract] OR rodent*[Title/Abstract])) AND ("isotopes"[MeSH Terms] OR "stable isotope*")) AND ("ecology"[MeSH Terms] OR Ecology[Title/Abstract] OR ecosystem*[Title/Abstract] OR "Trophic Niche"[Title/Abstract] OR "Trophic ecology"[Title/Abstract] OR "diet"[MeSH Terms] OR Diet[Title/Abstract] OR Dietary[Title/Abstract] OR "resource use"[Title/Abstract] OR "resource partitioning"[Title/Abstract]); Scopus \| TITLE-ABS-KEY ( ( Rodentia OR Rodents OR Rodent ) AND ( "stable isotope*" OR Stable W/3 Isotope* OR isotopes ) ) (ALL(rodentia) OR ALL(rodent)) AND (ALL("stable isotope") OR ALL("stable W/3 isotopes") OR ALL(isotopes)) AND ALL(ecology); Web of Science \| (ALL=(Rodentia OR Rodents OR Rodent)) AND ALL=( "stable isotopes" OR "Stable W/3 Isotopes" OR isotopes); SciELO \| ((((TS=(Rodentia)) OR TS=(rodents)) OR TS=(rodent)) AND TS=(stable isotopes)) OR TS=(isotopic analysis); Google Scholar \| "stable isotopes" AND Rodentia AND (ecology OR diet OR trophic OR niche) Two complementary search strategies were conducted in Scopus to maximize sensitivity and capture variations in indexing fields. The first strategy targeted title, abstract, and keywords (TITLE-ABS-KEY), while the second strategy used the ALL field to broaden retrieval. Note: Search strategies will be tailored to each database syntax. |
| Study records: |  |  |
| Data management | 11a | All records identified through the database searches will be imported into Zotero version 9.0 and the web-based software Rayyan, where duplicate records will be removed. |
| Selection process | 11b | Nine reviewers will screen randomized titles and abstracts in pairs according to the predefined eligibility criteria. Disagreements will be resolved through discussion and, when necessary, by consultation with an additional reviewer. Prior to formal screening, reviewers will pilot-test the eligibility criteria using a random sample of 100 records to ensure consistency in study selection. Inter-reviewer agreement may be assessed using percentage agreement.  Full-text screening will also be conducted by reviewer pairs, and disagreements will be resolved through discussion. Reasons for exclusion at the full-text stage will be documented and reported. The study selection process will be presented using the PRISMA-ScR flow diagram. |
| Data collection process | 11c | Data extraction will be conducted using a standardized charting form developed for this review. The charting form will be pilot-tested on a subset of included studies and refined iteratively prior to full data extraction to ensure consistency.  Extracted variables will include geographic location, ecosystem type, species, sample size, tissue type, isotope systems analyzed, analytical approach, ecological focus, study design and key findings. Additional variables may be incorporated during the charting process if considered relevant to the objectives of the review. If necessary, database searches may be rerun after a predefined period of one year to identify newly indexed studies relevant to the review. |
| Data items | 12 | N/A |
| Outcomes and prioritization | 13 | Data will be summarized descriptively using tables, figures and narrative synthesis. Included studies will be categorized according to isotope system, ecological application, ecosystem type, geographic distribution, tissue type, and methodological approach to identify major research trends and knowledge gaps. |
| Risk of bias in individual studies | 14 | Consistent with scoping review methodology, no formal critical appraisal of methodological quality or risk of bias will be conducted. The aim of this review is to map and characterize the available evidence regarding the application of stable isotope analysis in Rodentia ecology rather than to evaluate the quality or certainty of evidence. |
| Data synthesis | 15a | Included studies will be categorized according to isotope system, ecological application, ecosystem type, geographic distribution, tissue type, and methodological approach to identify major research trends and knowledge gaps. |
|  | 15b | Data will be summarized descriptively using tables, figures and narrative synthesis. |
|  | 15c | N/A |
|  | 15d | N/A |
| Meta-bias(es) | 16 | N/A |
| Confidence in cumulative evidence | 17 | N/A |

*** It is strongly recommended that this checklist be read in conjunction with the PRISMA-P Explanation and Elaboration (cite when available) for important clarification on the items. Amendments to a review protocol should be tracked and dated. The copyright for PRISMA-P (including checklist) is held by the PRISMA-P Group and is distributed under a Creative Commons Attribution Licence 4.0.**

**See: Shamseer L, Moher D, Clarke M, Ghersi D, Liberati A, Petticrew M, Shekelle P, Stewart L, PRISMA-P Group. Preferred reporting items for systematic review and meta-analysis protocols (PRISMA-P) 2015: elaboration and explanation. BMJ. 2015 Jan 2;349(jan02 1):g7647.*
